# Perceived Factors Influencing the Public Intention to Use E-Consultation: Analysis of Web-Based Survey Data

**DOI:** 10.2196/21834

**Published:** 2021-01-20

**Authors:** Miaojie Qi, Jiyu Cui, Xing Li, Youli Han

**Affiliations:** 1 School of Public Health Capital Medical University Beijing China

**Keywords:** China, e-consultation, perceived risk, surveys and questionnaires, technology acceptance model

## Abstract

**Background:**

Unbalanced distribution of medical resources is becoming a major challenge, particularly in the selection of doctors. e-Consultation could provide patients with more choices of doctors and break the constraints of time and space. However, the acceptance of e-consultation is still poor and the mechanism of adoption is unclear.

**Objective:**

The aim of this study was to identify the factors influencing the public intention to use e-consultation and explore the effect path of the factors and behavior intention.

**Methods:**

The hypotheses of our research model were developed based on the technology acceptance model and perceived risk theory. A web-based survey was conducted by an electronic questionnaire collection platform; this survey that consisted of a 29-item questionnaire with 5-point Likert scales was completed by 934 respondents. Structural equation modeling was used to analyze the data. Item evaluation and reliability, validity, path loading, goodness of fit, and multiple group analysis were used to check the moderation effects.

**Results:**

The standardized factor loadings of the items were between 0.551 and 0.873. The composite reliability of 9 constructs ranged from 0.706 to 0.840. The average variance extracted ranged from 0.387 to 0.640. The fitness indices showed that the collected data fitted well with the research model. Perceived usefulness was the strongest positive factor effecting behavior intention (β=.399, *P*<.001). Perceived ease of use had a positive effect on behavior intention but it was not statistically significant (β=.117, *P*=.07) and it had a positive effect on perceived usefulness (β=.537, *P*<.001). Perceived risk could be well explained by financial risk (β=.972, *P*<.001), privacy risk (β=.774, *P*<.001), social risk (β=.871, *P*<.001), time risk (β=.894, *P*<0.001), and psychological risk (β=.774, *P*<.001). Perceived risk had negative effects on perceived usefulness (β=–.375, *P*<.001) and behavior intention (β=–.297, *P*<.001). Personal innovativeness had a positive influence on perceived ease of use (β=.241, *P*<.001) and a slight effect on behavior intention (β=.124, *P=*.001). Age (*χ*^2^_58_=133.5, *P*<.001) and usage experience (*χ*^2^_58_=82.5, *P*=.02) had a slight moderation effect on the paths.

**Conclusions:**

Perceived usefulness and perceived risk have significant effects on public intention to use e-consultation. Therefore, platform and manufacturer must improve the function of e-consultation, which will promote the public intention to use e-consultation fundamentally. In order to control the perceived risk of public, government should play an important role in enforcing management of e-consultation markets and approving corresponding medical insurance policies. Besides, personal innovativeness had an effect on behavior intention. Moreover, the paths of factors had some heterogeneity among people with different characteristics. Therefore, it is necessary to adjust the strategies to fit more groups better.

## Introduction

### Background

In China, patients can directly go to tertiary referral hospitals to consult doctors, as primary care physicians do not have sufficient capacity to deal with complex diseases, which results in overloading of tertiary hospitals and increase in the unmet demands of patients [1,2]. With the outbreak of the COVID-19 pandemic, this overwhelming situation is becoming more prominent. e-Consultation provides a new way to solve this challenging situation between doctors and patients. e-Consultation—an innovative way to address the growing medical demand—allows users to overcome the barriers of space and time to have more possibilities of choosing doctors from the whole country, and it is becoming more widespread nowadays. e-Consultation can be classified into 2 types: inter-physician consultation and inter-patient-and-physician consultation [3]. In this study, we focused on the e-consultation between patients and health care providers and we did not involve telecare, telemonitor, and other eHealth. Specifically, users do not need to consult doctors in person and can obtain medical advice asynchronously after uploading personal illness information on the internet through video and text messaging to doctors [4,5].

Compared with face-to-face consultation, e-consultation has some natural advantages and unavoidable limitations. Specially, users only use words, pictures, and videos to communicate with doctors and are unable to receive a medical examination. Therefore, e-consultation is only used on nonurgent minor ailments now [6]. However, according to 2020 World Health Statistics, most patients develop common and chronic diseases, which means that e-consultation could meet great medical demands. In addition to medical advice on diagnosis and treatment regimen, e-consultation could provide patients with timelier and more convenient care [7,8], reduce cost for patients [7,9,10], and improve equitable access for underserved patients and to specialist care [11,12]. For the health system, e-consultation could improve the efficiency of referrals and face-to-face consultation [13-17] and improve the quality of health care [13,18]. Therefore, e-consultation might be a potential solution for major challenges that our health care system faces today [12]. However, many patients are unwilling to use e-consultation even if their illnesses are not serious because they prefer to see doctors in person [19]. A study showed that the average workload of doctors providing web-based health care services was 0.38 patients in China [20] and the situation that people lack awareness of e-consultation exists in a developed country too [21]. For the large part, users are unwilling to believe in the judgments of the doctors in web-based health care services without seeing doctors and without undergoing a medical examination [22]. Our previous survey also found that patients with prior experience of using e-consultation went to the hospital later for the same disease because they wanted to check if the judgement of the doctors providing web-based health care services was accurate. There are great risks perceived by patients if they follow the e-consultation judgement completely. Therefore, many people stated that with the help of their primary care providers, they can use e-consultation better in order to avoid mistakes [23].

As mentioned above, there are many researches focused on the clinical and socioeconomic effects of e-consultation, but these researches have not explored the process or the acceptance of e-consultation or the barriers and the promoters of e-consultation [24]. The content of e-consultation is significantly different from the other functions of eHealth; therefore, we cannot simply apply the usage mechanism of eHealth into e-consultation. This study can fill this gap effectively. The acceptance of e-consultation is a matter of accepting medical information technology, and the technology acceptance model (TAM) explains the acceptance behavior of information communication technology for individuals well [25]. However, our preliminary research and field investigation showed that perceived risk was a significant factor influencing usage behavior, which was reported in many studies as well. However, perceived risk is always taken as a simple dimension in prior researches, which lead to the lack of specific and accurate guiding effects on reality. This research aims to further decompose the perceived risk dimension comprehensively. Therefore, combining TAM and perceived risk, we reconstructed a new model to explore the acceptance mechanism of e-consultation, and we hope this research would help governments and providers make effective and efficient intervention strategies.

### Theoretical Background

#### TAM

The TAM was proposed by Davis based on the previous theories. TAM focuses on an individual’s intention to accept information technology. In TAM, perceived usefulness is defined as the extent to which people believe apps would help them perform their job better. Perceived ease of use is defined as the extent to which people believe using apps would be free of effort. Perceived usefulness and perceived ease of use are the 2 main elements that have influence on the intention to use, and sufficient intention leads to actual usage behavior [25]. TAM has been successfully adapted in many eHealth [26], mobile health [27], mobile management systems [28,29], and web-based medical websites [30].

#### Perceived Risk Theories

A lot of researches show that perceived risk is a key factor that influences people to use medical innovations [28,31-33]. In the medical field, the public always makes medical decisions uncertainly due to information asymmetry, especially when using some emerging medical products and functions. e-Consultation has not really realized the maturity of technology and the stability of the service mode, which aggravates the uncertainty. As shown in the research that even if patients reported satisfaction and acceptance of e-consultation, they did not express strong interests in participating in this interaction because of medical responsibility and accuracy of disease description [34]. Therefore, we take perceived risk as one of the core dimensions of this study and integrate it with TAM.

Originally, perceived risk illustrates the mechanism of people for accepting new brands in the commercial market. It is a sense of uncertainty caused by consumers’ inability to predict the outcome of their purchases. The components of perceived risk includes performance risk, physical risk, financial risk, social risk, and psychological risk [35]. With the development of the perceived risk theory, more components are added into the construct, including time risk [36] and privacy risk [37]. Perceived risk theory holds the view that people try to minimize the perceived risk of behavior rather than to maximize the perceived benefit when making consumption decisions [38]. In our study, performance risk is defined as the possibility of e-consultation not performing as it is designed [35,39]. Physical risk is the chances that e-consultation could result in delays in treatment or in misdiagnoses [35]. We can find that delaying treatment or a misdiagnosis means performing out of control; therefore, we just need to keep one factor between performance risk and physical risk. Financial risk and time risk refer to the possibility that users may face loss of money and time when using e-consultation [35,39]. Social risk is the chances that the use of e-consultation would affect the way others think of the users [35,39]. Psychological risk is the chance that e-consultation would not fit in well with users’ self-image or self-concept [35,39]. Privacy risk is the potential loss of control over personal information [35,39].

#### Personal Innovativeness

Personal innovativeness is defined as the degree to which a person is relatively willing to adopt e-consultation in this study [40]. The relationship between technology and the degree of receptiveness to innovation determines how quickly a person adopts information and communications technology [41]. Personal innovativeness can explain the individual differences in their perception of e-consultation advantages and risks. Individuals with higher innovativeness prefer change and tend to gather more information of the technical products. The positive attitude of the innovator toward products would be promoted by the increased interaction with products, which makes them pay more attention to the advantages of technical products and not worry about products working in the designed way [42]. This viewpoint has also been tested in several researches of mobile health adoption [27,43].

### Research Model and Hypotheses

The public can choose any registered doctor on the e-consultation platform with a limited cost. The platforms provide users with all kind of hospital departments with different service levels from different regions. Thus, the public have more access for better consultation services. Through e-consultation, users can receive valuable suggestions easily and quickly. After obtaining enough suggestions, they are able to make and follow health decisions better. These functions of e-consultation are attractive to the users. Besides, if it is easy to learn how to use e-consultation, it means that the public will accept e-consultation easier without much effort. Thus, we propose the following hypotheses based on TAM:

Hypothesis 1: Perceived usefulness will influence behavior intention positively.

Hypothesis 2: Perceived ease of use will influence behavior intention positively.

Hypothesis 3: Perceived ease of use will influence perceived usefulness positively.

If e-consultation provides incorrect suggestions, users would be delayed in accepting correct treatment or they may receive wrong treatment. Loss of performance means a loss of health. Unlike the common consumer behavior, performance risk and physical risk are always perceived by the public together. Therefore, physical risk could be absorbed into performance risk. In the TAM, perceived usefulness reflects the functions of e-consultation as well. High levels of risk perceived by people means that they have a suspicion on the usefulness of e-consultation. Therefore, it is unnecessary to integrate performance risk and physical risk into the model again. Besides, since e-consultation needs users to submit symptoms, medical records, and other personal information, the operation of e-consultation would be a new challenge for the user. Thus, we propose the following hypotheses:

Hypothesis 4a: Financial risk is a component of perceived risk of using e-consultation.

Hypothesis 4b: Privacy risk is a component of perceived risk of using e-consultation.

Hypothesis 4c: Social risk is a component of perceived risk of using e-consultation.

Hypothesis 4d: Time risk is a component of perceived risk of using e-consultation.

Hypothesis 4e: Psychological risk is a component of perceived risk of using e-consultation.

Hypothesis 5: Perceived risk will influence perceived usefulness negatively.

Hypothesis 6: Perceived risk will influence perceived ease of use negatively.

Hypothesis 7: Perceived risk will influence behavior intention negatively.

e-Consultation as a combination of information technology and medical services subverts the traditional concept of consultation. Therefore, if people have better innovativeness, they are willing to adopt all kinds of new information technology, including e-consultation. Some other researches show that personal innovativeness also has a direct effect on perceived ease of use. Because people with high level of innovativeness have richer experience in using emerging products, they would think the operation of e-consultation is less difficult. Thus, we propose the following hypotheses:

Hypothesis 8: Personal innovativeness will influence perceived ease of use positively.

Hypothesis 9: Personal innovativeness will influence behavior intention positively.

**Figure 1:**
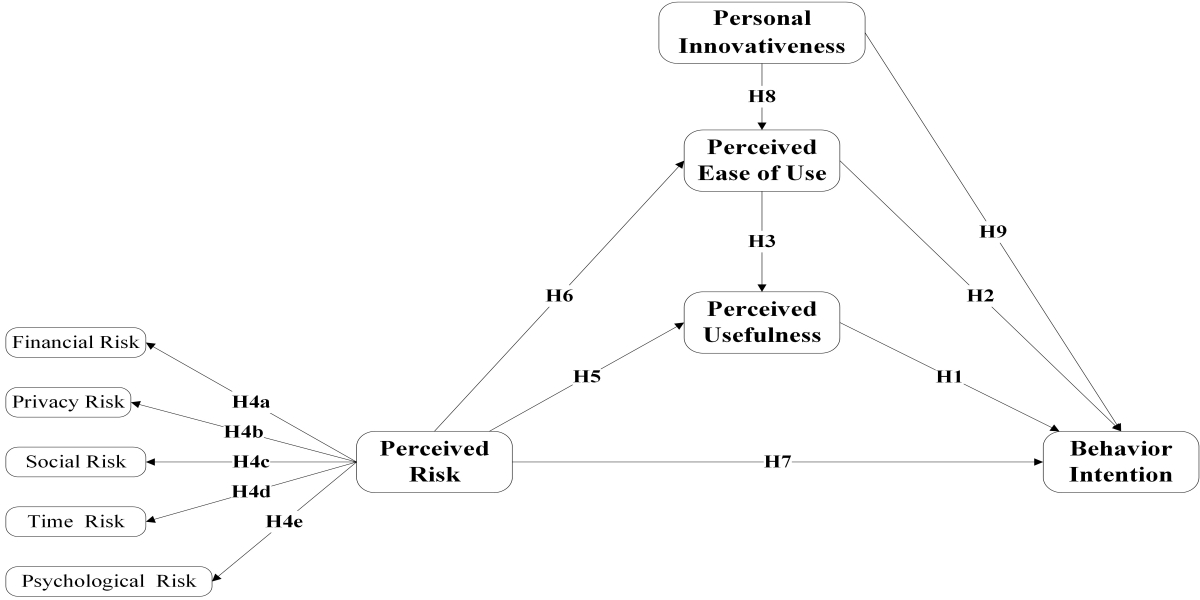
Research model based on the technology acceptance model and perceived risk theory. Personal innovativeness had effects on behavior intention and perceived ease of use. H: hypothesis.

Overall, the research model is showed in Figure 1.

### Aim of This Study

The objective of this study was to investigate people’s actual usage of e-consultation and their characteristics. Moreover, based on the TAM integrating with perceived risk and personal innovativeness theory, a questionnaire survey was used to explore the relationships and paths of the factors that influence people’s intention to use e-consultation.

## Methods

### Study Design

All survey items were adopted from previous studies related to eHealth and health information technology. The first version of the questionnaire was directly translated from English to Chinese by a group of researchers. Items were reasonably changed to adapt to the e-consultation. Then, the second version of the questionnaire was completed after 2 rounds of experts’ discussions on the first questionnaire. The experts consisted of 2 college professors, 7 staffs from an eHealth company, and 7 doctors with e-consultation using experience. Some items were added or removed or replaced according to the suggestions of experts. The third modification of the questionnaire was completed after a preliminary survey of 222 students majoring in health management from Capital Medical University. Some items were removed or changed to ensure the reliability and validity of the questionnaire. In the end, back translation was performed from Chinese to English by another qualified translator. The final items (Table 1, [44-46]) were measured with a 5-point Likert scale ranging from “strongly disagree” (1) to “strongly agree” (5). The final questionnaire consisted of 2 parts. The first part was the demographic information of the respondents. The second part, which includes the items for constructs, was designed to measure the respondents’ perception on each item.

**Table 1:** Measurement items of the constructs.

Construct		Item
**PU**^a^ [25,44,45]		
	PU1	Using e-consultation would make it easier to consult a specialist or a certified doctor.
	PU2	Using e-consultation enables me to understand my disease and treatment recommendation more quickly.
	PU3	Using e-consultation facilitates complete communication with doctor.
	PU4	Using e-consultation enables me to know more about disease prevention and management.
	PU5	Using e-consultation enables me to make better treatment-related decisions.
	PU6	I find it easy to obtain information on e-consultation.
**PEU**^b^ [25,44,45]		
	PEU1	Learning to use e-consultation is easy for me.
	PEU2	In e-consultation, my doctor talks to me clearly and helps me understand my situation appropriately.
	PEU3	Using e-consultation would not require much mental effort.
	PEU4	It is easy for me to become skillful at using e-consultation.
**FR**^c^ [31,35,39]		
	FR1	e-Consultation is not effective and is a waste of money.
	FR2	e-Consultation may make me spend extra money in case of a misdiagnosis, leading to delayed correct treatment.
	FR3	Using e-consultation may lead to potential fraud.
**PRR**^d^ [35,39]		
	PRR1	After using e-consultation, my personal information may be leaked.
	PRR2	After using e-consultation, my personal information may be used without my knowledge.
	PRR3	After using e-consultation, my illness information may be found by others around me.
**SR**^e^ [31,35,39]		
	SR1	If I use e-consultation, it would negatively affect the way others think of me.
	SR2	If I use e-consultation, my friends and relatives would think less highly of me.
**TR**^f^ [31,39]		
	TR1	e-Consultation may be a waste of time because it is not effective.
	TR2	e-Consultation may be a waste of time because of wrong diagnoses or treatments.
**PSR**^g^ [31,35,39]		
	PSR1	e-Consultation is not my traditional way to consult doctors, which would lead to psychological issues
	PSR2	I am unable to communicate with doctors face-to-face thereby leading to psychological issues
	PSR3	I am worried that I cannot describe my disease symptoms correctly when using e-consultation.
**PI**^h^ [40,45,46]		
	PI1	I often follow new information technologies with interest.
	PI2	If I hear about a new information technology, I would look for ways to experiment with it.
	PI3	Among my peers, I am usually the first to try out new information technologies.
**BI**^i^ [25]		
	BI1	I intend to use e-consultation.
	BI2	I intend to use more e-consultation.
	BI3	I predict that I will use e-consultation.

^a^PU: perceived usefulness.

^b^PEU: perceived ease of use.

^c^FR: financial risk.

^d^PRR: privacy risk.

^e^SR: social risk.

^f^TR: time risk.

^g^PSR: psychological risk.

^h^PI: personal innovativeness.

^i^BI: behavior intention.

### Data Collection

With the development of information technology, internet protocol restriction, and real-name system, the data quality of web-based surveys meets the requirements of scientific researches. As mentioned above, the users of e-consultation are mainly concentrated in young and middle-aged groups, and the middle-aged group is more willing to accept web-based questionnaires. Therefore, a web-based survey was conducted by Sojump in this research. Sojump is an e-survey company [47], which has 2.6 million samples with all kinds of social demographic characteristics, and unqualified objects can be excluded based on the purpose of the study. The questionnaire was announced on Sojiangwang [48] until the required population was reached. The Sojiangwang is a platform belonging to Sojump, in which all kinds of people can register in. The Sojiangwang asks every registrant to upload the real identity information and audit the identity information. In this platform, the registrant can see all the questionnaires when they meet the included standard of the questionnaires. All different questionnaires would be named with a unified format: “questionnaire + number.” In order to ensure the quality of the survey, Sojump uses a series of logical and common sense items to eliminate the halfhearted respondents, and 828 respondents were excluded by this way in our study. Besides, we also designed 2 items to screen the poor-quality questionnaires. The first item listed in the demographic information survey is “e-Consultation could provide surgical and pathological examination services” and the respondents were asked to choose “disagree.” The second item listed in the construct is “Now, e-consultation could provide diagnosis and treatment for all kind of diseases” and the respondents were asked to choose “strongly disagree.”

**Figure 2:**
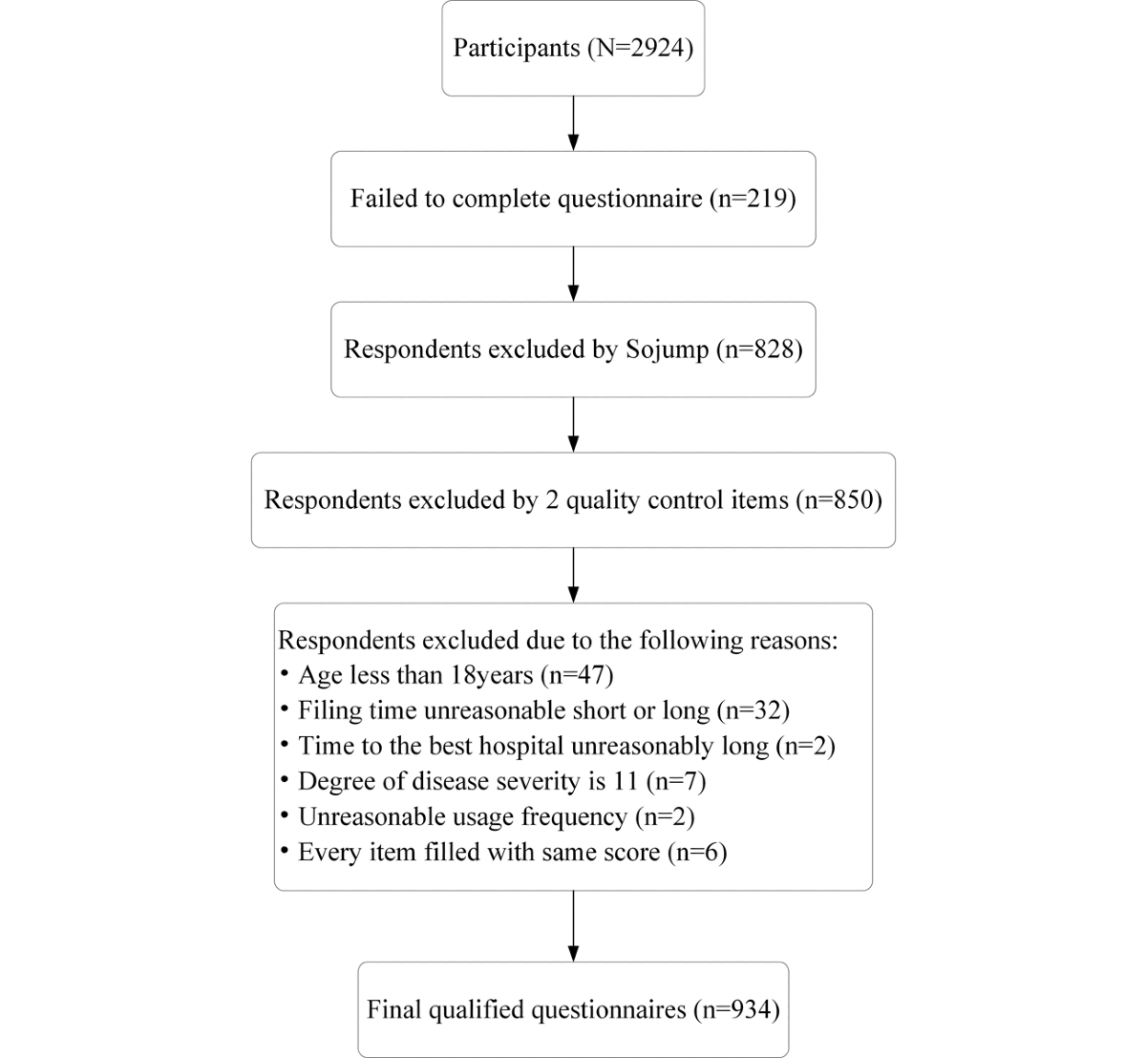
Sampling procedure.

At the beginning of the electronic questionnaire, the following information was given first: the purpose of the questionnaire, information and instructions regarding the questionnaire, assurance of proper handling of personal information, and the name of the research institution. The questionnaire link provided on the website (Sojiangwang) could not be copied. After users filled in the questionnaire through the link, the link was removed from the list and could not be used repeatedly. We have reported the results of this survey following the CHERRIES (Checklist for Reporting Results of Internet E-Surveys) checklist, which can be found in Multimedia Appendix 1. The data were collected from March 30, 2020 to April 4, 2020. A total of 2924 participants were involved in this survey, and 934 respondents’ questionnaires reached the inclusion criteria of the survey (Figure 2). This study was approved by the ethics committee of the Capital Medical University (number Z2019SY017).

As shown in Table 2, female respondents were more than male respondents. Most respondents were between 18 and 45 years of age (861/934, 92.2%). The education level of the respondents was good because only 21.7% (203/934) of the respondents had a lower level of education than bachelor’s degree. Most respondents were from the more developed eastern region (559/934, 59.9%) and urban region (797/934, 85.4%). The level of income and the access to medical resources were relatively average. Approximately 67.5% (630/934) of the respondents had used e-consultation, of which 80.1% (505/630) had used e-consultation 5 times or less last year. The aims of using e-consultation include helping themselves (352/630, 55.9%) and others (278/630, 44.1%). Both serious and minor diseases could be the subject of e-consultation.

**Table 2:** Geographical characteristics of the respondents (N=934).

Characteristics		n (%), Value
**Gender**		
	Male	379 (40.6)
	Female	555 (59.4)
**Age (years)**		
	18-25	239 (25.6)
	26-35	463 (49.6)
	36-45	159 (17.0)
	>46	73 (7.8)
**Education**		
	Middle school or lower	13 (1.4)
	High school	52 (5.6)
	Three-year college	138 (14.7)
	Bachelor	660 (70.7)
	Master or higher	71 (7.6)
**Residence**		
	Rural	137 (14.6)
	Urban	797 (85.4)
**Location**		
	Eastern	559 (59.9)
	Midregion	164 (17.5)
	Western	152 (16.2)
	Northern	59 (6.3)
**Average annual income (¥, US $1=¥6.475)**		
	0-10,000	297 (31.8)
	11,000-20,000	348 (37.3)
	21,000-30,000	174 (18.6)
	>30,000	115 (12.3)
**Time to the best hospital in district and county (minutes)^a^**		
	1-10	281 (30.1)
	11-20	289 (30.9)
	21-30	227 (24.3)
	>30	137 (14.7)
**Usage experience**		
	Used	630 (67.5)
	Unused	304 (32.5)
**Usage frequency last year**		
	0-2	215 (34.1)
	3-5	290 (46.0)
	>5	125 (19.9)
**Use e-consultation for whom**		
	Myself	352 (55.9)
	Others	278 (44.1)
**Disease severity^b^**		
	1-5	337 (53.5)
	6-10	293 (46.5)

^a^Measures the accessibility of high quality medical resources.

^b^Users themselves assessed the severity of last disease consulted by e-consultation using a 5-point Likert scale ranging from “strongly not serious” (1) to “strongly serious” (10).

### Data Analysis

SPSS 20.0 (IBM Corp) was used to analyze the descriptive statistics of respondents’ demographic characteristics and the Cronbach α of the constructs. Amos 24.0 (IBM Corp) was used to evaluate items, measurements, and structural models. Confirmatory factor analysis of the measurement model was used to evaluate the structural model’s path effects, significance, goodness of fit, and moderation effects. Composite reliability and average variance extracted were adopted to evaluate construct reliability and validity.

## Results

### Measurement Model Testing

The results of reliability and validity are shown in Table 3. The composite reliability, Cronbach α of construct, was greater than the recommended value of .7, and except for financial risk, perceived usefulness, and perceived ease of use, the average variance extracted of constructs was higher than 0.5 [49]. In order to assure the availability of the model, we excluded the low loading items (PU3, PU4) to construct model 2 for testing the result of model 1. We found that although the average variance extracted was promoted a little in model 2, the model fit indices had no substantial improvement (Table 3). Further, the path effects had no substantive difference between model 1 and model 2. Besides, some researches showed that all factors fulfilled a weak or strong validity because factor loadings were statistically significant and the coefficients of path were substantial [50]. Therefore, it was reasonable to accept the results of model 1. As shown in Table 4, the collected data fit well with the research model [51]. The *χ*^2^/df (1111.9/363) of model 1 was 3.1 and was lower than 5. The root mean square error of approximation was 0.047 and was lower than 0.05. The goodness of fit index, comparative fit index, normed fit index, Tucker-Lewis index, and incremental fit index were greater than 0.9 and the adjusted goodness of fit index was 0.9.

**Table 3:** Item loading and validity.

Construct/Item		Factor loading		Cronbach α		Composite reliability		Average variance extracted	
		Model 1	Model 2	Model 1	Model 2	Model 1	Model 2	Model 1	Model 2
**PU^a^**				.754	.740	0.790	0.740	0.387	0.416
	PU1	0.664	0.665						
	PU2	0.652	0.651						
	PU3	0.577	—^b^						
	PU4	0.551	—						
	PU5	0.640	0.639						
	PU6	0.640	0.625						
**PEU^c^**				.743	.743	0.747	0.748	0.425	0.426
	PEU1	0.623	0.629						
	PEU2	0.665	0.658						
	PEU3	0.645	0.646						
	PEU4	0.673	0.676						
**FR^d^**				.744	.744	0.745	0.745	0.495	0.494
	FR1	0.648	0.647						
	FR2	0.734	0.734						
	FR3	0.725	0.725						
**PRR^e^**				.829	.829	0.840	0.840	0.640	0.640
	PRR1	0.867	0.867						
	PRR2	0.873	0.874						
	PRR3	0.638	0.637						
**SR^f^**				.702	.702	0.706	0.706	0.547	0.547
	SR1	0.789	0.789						
	SR2	0.687	0.687						
**TR^g^**				.761	.761	0.761	0.761	0.614	0.614
	TR1	0.786	0.786						
	TR2	0.781	0.781						
**PSR^h^**				.754	.754	0.768	0.768	0.529	0.529
	PSR1	0.813	0.813						
	PSR2	0.766	0.765						
	PSR3	0.583	0.583						
**PI^i^**				.745	.745	0.753	0.753	0.509	0.509
	PI1	0.575	0.575						
	PI2	0.792	0.792						
	PI3	0.754	0.754						
**BI^j^**				.790	.790	0.789	0.789	0.556	0.556
	BI1	0.768	0.768						
	BI2	0.768	0.768						
	BI3	0.698	0.697						

^a^PU: perceived usefulness.

^b^Not available.

^c^PEU: perceived ease of use.

^d^FR: financial risk.

^e^PRR: privacy risk.

^f^SR: social risk.

^g^TR: time risk.

^h^PSR: psychological risk.

^i^PI: personal innovativeness.

^j^BI: behavior intention.

**Table 4:** Research model fit.

Fit index	Value (*χ*^2^/*df)*	GFI^a^	AGFI^b^	RMSEA^c^	CFI^d^	NFI^e^	TLI^f^	IFI^g^
Recommended value	<5	>0.9	>0.9	<0.05	>0.9	>0.9	>0.9	>0.9
Value in model 1	3.1 (1111.9/363)	0.917	0.900	0.047	0.932	0.903	0.924	0.933
Value in model 2	3.2 (1002.4/310)	0.919	0.901	0.049	0.934	0.907	0.925	0.934

^a^GFI: goodness of fit index.

^b^AGFI: adjusted goodness of fit index.

^c^RMSEA: root mean square error of approximation.

^d^CFI: comparative fit index.

^e^NFI: normed fit index.

^f^TLI: Tucker-Lewis index.

^g^IFI: incremental fit index.

### Structural Model Testing

The judgments of hypotheses based on the SEM results are shown in Table 5. The judgments of model 1 and model 2 exhibited the same results and the standardized factor loadings of path were very closed. Perceived ease of use had no statistically significant effect on behavior intention (β=.117, *P*=.07; β1=.104, *P*=.13). Perceived usefulness had a positive effect on behavior intention (β=.399, *P*<.001; β1=.431, *P*<.001) and was the strongest positive factor of behavior intention. Perceived ease of use had a positive effect on perceived usefulness (β=.537, *P*<.001; β1=.530, *P*<.001). Perceived risk could be well explained by financial risk (β=.972, *P*<.001; β1=.973, *P*<.001), privacy risk (β=.774, *P*<.001; β1=.774, *P*<.001), social risk (β=.871, *P*<.001; β1=.870, *P*<.001), time risk (β=.894, *P*<.001; β1=.894, *P*<.001), and psychological risk (β=.774, *P*<.001; β1=.774, *P*<.001). Among the components, the effect of financial risk was the strongest and that of social risk was the weakest. Perceived risk had negative effects on perceived usefulness (β=–.375, *P*<.001; β1=–.399, *P*<.001) and behavior intention (β=–.297, *P*<.001; β1=–.275, *P*<.001). Personal innovativeness had a positive influence on perceived ease of use (β=.241, *P*<.001; β1=.242, *P*<.001). Compared with other factors, personal innovativeness had a slight effect on behavior intention (β=.124, *P*=.001; β1=.123, *P*=.001).

**Table 5:** Results of hypothesis testing.

Hypothesis	Path	β^a^	*P* value	Judgement of model 1	β1^b^	*P* value	Judgement of model 2
H1	PU^c^→BI	.399	<.001	Accepted	.431	<.001	Accepted
H2	PEU^d^→BI	.117	.07	Rejected	.104	.13	Rejected
H3	PEU→PU	.537	<.001	Accepted	.530	<.001	Accepted
H4a	PR^e^→FR^f^	.972	<.001	Accepted	.973	<.001	Accepted
H4b	PR→PRR^g^	.774	<.001	Accepted	.774	<.001	Accepted
H4c	PR→SR^h^	.537	<.001	Accepted	.536	<.001	Accepted
H4d	PR→TR^i^	.894	<.001	Accepted	.894	<.001	Accepted
H4e	PR→PSR^j^	.871	<.001	Accepted	.870	<.001	Accepted
H5	PR→PU	–.375	<.001	Accepted	–.399	<.001	Accepted
H6	PR→PEU	–.491	<.001	Accepted	–.488	<.001	Accepted
H7	PR→BI	–.297	<.001	Accepted	–.275	<.001	Accepted
H8	PI^k^→PEU	.241	<.001	Accepted	.242	<.001	Accepted
H9	PI→BI^l^	.124	.001	Accepted	.123	.001	Accepted

^a^β: standardized factor loading of model 1.

^b^β1: standardized factor loading of model 2.

^c^PU: perceived usefulness.

^d^PEU: perceived ease of use.

^e^PR: perceived risk.

^f^FR: financial risk.

^g^PRR: privacy risk.

^h^SR: social risk.

^i^TR: time risk.

^j^PSR: psychological risk.

^k^PI: personal innovativeness.

^l^BI: behavior intention.

### Moderation Effect Testing

We further tested the moderating effects of geographical characteristics by multiple-group analysis [52,53]. In order to simplify the data analysis, the total sample was reclassified into 2 subgroups (Table 6). First, to screen the factors with moderation effects from characteristics, we constrained the measurement weights, structural weights, structural covariances, structural residua, and measurement residua of the subgroup model to construct parameter constraints models. If the results of the constraints model and the unconstrained model were significantly different, it indicated that the paths between subgroups are the factors that might have a moderation effect. As showed in Table 6, age (*χ*^2^_58_=133.5, *P*<.001), income (*χ*^2^_58_=85.6, *P*=.01), and usage experience (*χ*^2^_58_=82.5, *P*=.02) might have moderation effects.

**Table 6:** Dichotomous geographical characteristics of the respondents (N=934).

Characteristics		n (%), Value	*χ* ^2^ *(df)*	*P* value
**Gender**			43.3 (58)	.93
	Male	379 (40.6)		
	Female	555 (59.4)		
**Age (years)**			133.5 (58)	<.001
	18-30	464 (49.7)		
	>30	470 (50.3)		
**Education**			25.7 (58)	>.99
	Three-year college or lower	203 (21.7)		
	Bachelor or high	731 (78.3)		
**Residence**			47.8 (58)	.83
	Rural	137 (14.6)		
	Urban	797 (85.4)		
**Location**			38.2 (58)	.98
	Eastern	559 (59.9)		
	Not eastern	375 (40.1)		
**Income (¥, US $** **1** **=¥6.475)**			85.6 (58)	.01
	0-10,000	297 (31.8)		
	>10,000	637 (68.2)		
**Time to the best hospital** **(minutes)**			35.7 (58)	.99
	1-20	570 (61.0)		
	>21	364 (39.0)		
**Usage experience**			82.5 (58)	.02
	Used	630 (67.5)		
	Unused	304 (32.5)		
**Usage frequency last year**			49.4 (58)	.78
	0-2	215 (34.1)		
	>2	415 (65.9)		
**Use e-consultation for whom**			44.1 (58)	.91
	Myself	352 (55.9)		
	Others	278 (44.1)		
**Disease severity**			70.5 (58)	.13
	1-5	337 (53.5)		
	6-10	293 (46.5)		

Second, we estimated the path loadings and the critical ratios for differences of each subgroup (Table 7). If the absolute value of the critical ratio was lower than 1.96, there would be a significant difference between the paths of the 2 subgroups. Compared with the older subgroup (β_age2_=.235, *P*=.02), it is estimated that perceived usefulness has more positive effect on behavior intention in the younger subgroup (β_age1_=.537, *P*<.001). The path loading of hypothesis 9 was not significant in the older subgroup (β_age1_=.054, *P*=.34). Income had no significant moderation influence on the research model. Besides, the usage experience only had some influence on path coefficient. It is shown that the path loading of perceived ease of use to behavior intention has a significant difference, but the coefficients were very close (β_experience1_=.532; β_experience2_=.534). The path loading of personal innovativeness to perceived ease of use in the used group (β_experience1_=.149, *P*=.008) was lower than that of the unused group (β_experience1_=.327, *P*<.001). We found that there was no substantial difference in the usage mechanism whether or not the public used e-consultation.

**Table 7:** Multiple group analysis.

Hypothesis (H)	β_age1_^a^	β_age2_^b^	CR^c^	β_income1_^d^	β_income2_^e^	CR	β_experience1_^f^	β_experience2_^g^	CR
H1	.537***	.235 (.022)	–2.221**	.346***	.432***	0.203	.498***	.345***	0.02
H2	.09 (.328)	.172 (.057)	0.556	.115 (.251)	.111 (.193)	–0.030	.045 (.630)	.137 (.162)	0.629
H3	.464***	.587***	1.025	.554***	.538***	0.571	.532***	.534***	–2.043**
H4a	.957***	.987***	—^h^	.942***	.980***	—	.980***	.944***	—
H4b	.741***	.795***	–0.226	.754***	.766***	–1.338	.769***	.688***	0.364
H4c	.442***	.630***	0.511	.515***	.512***	–1.729	.557***	.376***	0.625
H4d	.847***	.943***	–0.694	.800***	.932***	–1.507	.874***	.851***	1.087
H4e	.842***	.899***	–1.274	.821***	.880***	–1.632	.880***	.745***	0.787
H5	–.391***	–.367***	–0.294	–.297***	–.390***	–0.252	–.393***	–.314***	0.306
H6	–.534***	–.456***	0.022	–.408***	–.501***	0.538	–.490***	–.318***	–0.513
H7	–.221 (.004)	–.371***	–1.704	–.323***	–.272***	1.386	–.247***	–.255 (.001)	–1.193
H8	.203 (.002)	.284***	1.831	.258 (.002)	.229***	–0.704	.149 (.008)	.327***	2.785**
H9	.054 (.344)	.186***	2.159**	.197 (.004)	.097 (.039)	–1.47	.125 (.016)	.127 (.063)	0.594

^a^β_age1_: standardized factor loading of age from 18 years to 30 years.

^b^β_age2_: standardized factor loading of age over 30 years.

^c^CR: critical ratios for differences.

^d^β_income1_: standardized factor loading of income below ¥100,000 per year; US $1=¥6.475.

^e^β_income2_: standardized factor loading of income over ¥100,000 per year.

^f^β_experience1_: standardized factor loading of the used.

^g^β_experience1_: standardized factor loading of the unused.

^h^Not available due to fixed parameter.

**P<.05.

***P<.001.

## Discussion

### Principal Results

Our study found that perceived usefulness is one of the most important determinants of individuals’ intention to use e-consultation, which is similar to that reported in most related studies on the acceptance of information communication technology [27,29,30]. Even in subgroups with different characteristics, the direction and significance of path loading were not changed. Our results indicate that promoting the function of e-consultation is a key to attract the public to use it because the higher perceived usefulness means the public have more trust in the ability and integrity of doctors and platforms [24]. Compared with face-to-face consultation, e-consultation could only be used to diagnose common and chronic diseases lacking necessary medical examinations and the supporting treatment system, but the text suggestions from the specialists are still important for the public. In particular, in some special cases (eg, COVID-19 pandemic), e-consultation could not only achieve the goal of public isolation but also meet the patients’ demand of medical services. Although e-consultation cannot be a complete substitute for face-to-face consultations, it may serve as an entry level consultation after integrated into the face-to-face consultation [54]. In addition to the service ability of e-consultation, technical difficulties, including substandard signal construction, virtual device, and video equipment, would significantly weaken people’s evaluation on the usefulness of e-consultation [55]. Therefore, while improving the functionality, providers should also pay attention to improve the facilitation condition of e-consultation.

Different from the related researches, our research shows that perceived ease of use has no effect on the behavior intention but it has a strong effect on perceived usefulness. Normally, since the information communication technology products are used in the professional field, learning to use these products is a challenge for users. However, e-consultation just needs users to interact with doctors on the internet by using a personal computer or a smartphone. With the popularity of smartphones in China and worldwide [56], it is reasonable to believe that the public can easily learn how to operate e-consultation; therefore, the ease of use no longer plays a role in the promotion. As shown in a survey of 947 respondents, less than 20% of the people think that the reason they do not use e-consultation is that they are not skillful enough to complete the operation [57]. Another possible explanation is related to the characteristics of the respondents. Perceived ease of use comprises ease of operation, understanding, and expression. In our survey, most of the respondents had high educational backgrounds and were young, which leads to a stronger understanding ability, thereby leading to no significant relationship between perceived ease of use and behavior intention.

Personal innovativeness has a direct effect on perceived ease of use and behavior intention, which is consistent with the findings of a previous study [45]. Although more and more high-tech products are emerging, the public are always keen to try the popular products rather than the new ones. Therefore, it is essential to strengthen the publicity of e-consultation for the public. Of course, formatting innovativeness is a complex and long process [58]; therefore, finding the innovative individual might be a better choice. In the promotion of e-consultation, e-consultation providers should offer advanced services for the innovative and stable services for the common.

Perceived risk has a significantly negative effect on behavior intention. Because of health issues, the public would take risks more seriously and perform risk aversion [59]. Uncertainty and information asymmetry are typical features of medical services, which always leads patients with common diseases to fail in selecting the most effective services (primary health care). They would prefer to go to a tertiary hospital for the minimization of medical risk instead of the maximization of utility [60]. Different from other results that risk influence intention [29,38] or attitude [37] directly, our results show that perceived risk weakens not only behavior intention but also perceived usefulness and perceived ease of use directly. It shows that if we do not control the risk of e-consultation, even if e-consultation could provide services for more diseases, the public would reduce the evaluation and the intention of e-consultation. In addition, perceived usefulness contains performance risk and physical risk and perceived ease of use reflects the risk of operation. It indicates that performance risk, physical risk, and operation risk are components of perceived risk, which is consistent with the theoretical hypothesis and our previous surveys. However, perceived ease of use has no effect on behavior intention. Therefore, we need to take note that ease of operation may not promote the usage intention, but the difficulty of operation may reduce the intention.

There are many studies on the barriers in e-consultation, but these only explored the objective and external factors, for example, signal coverage, equipment, and characteristics of the patients. Even if some qualitative studies ask users’ subjective evaluation, the final results are not comprehensive [61]. Our study made up for some gaps in these researches and found that among the other components, financial risk and time risk were the most considered by people. In China, the e-consultation platforms provide free and paid consultation services for users. The choices of doctors and number of questions would be limited in the free services and the cost of paid services cannot be submitted to medical insurance. Therefore, if e-consultation is not effective, the cost and time of using e-consultation will be wasted altogether. Compared with the indirect costs [62] saved by e-consultation, the opportunity cost of e-consultation is more valued by the public. Besides, not all patients believed that e-consultations could play the role in reducing the time to access specialists’ advice [63]. Therefore, it is important to strengthen the connection between e-consultation and offline treatment and include the cost of e-consultation into the medical insurance system. These are the 2 keys to promote e-consultation use.

Many studies have found that the key barriers of using e-consultation for patients are privacy concerns and security of their data [5,19,64]. The path of perceived risk and privacy risk showed that providers should strengthen the construction of e-consultation information systems. Privacy disclosure has been a big problem in the medical field [65,66]. Users worry about not only the illegal disclosure but also the exposure to their family members with some special diseases such as mental illness [67]. We should appeal to the government to make related laws and strengthen the supervision of the operation of the e-consultation platform, and then, the public would upload their personal medical information during e-consultation.

e-Consultation, a new consultation model, has been in China for less than 20 years, and the public have little detailed knowledge about it; therefore, the public cannot get used to this kind of non–face-to-face consultation quickly, which has aroused the public’s attention to psychological risk. Besides, body language is often accompanied by patients’ expression, but the text-based e-consultation cannot reveal the body language, which can easily cause anxiety about the incomplete expression for patients [68]. Real-time video calls could alleviate this problem to some extent, but it is not applicable to all patients because of limitations in different video equipment. Of course, since the public with general health literacy are often unable to describe the uncommon disease symptoms and feelings correctly [69], they would worry about their medical behavior in e-consultation. Therefore, it would be necessary to improve the public’s health literacy to decrease the psychological risk. In fact, the lack of people’s health literacy is a long-standing problem and it is difficult to be overcome completely [70]. We need to cooperate with certain auxiliary ways to assist the public to use e-consultation, among which keeping a special receptionist [71] may be a good solution.

Although social risk is only a minor component of perceived risk, we need to improve the awareness of e-consultation among the public to help them understand it correctly. As mentioned above, the public know less about e-consultation; therefore, there is no effective consensus on e-consultation in the society. Some researches show that the most prominent reason for nonuse of e-consultation is that the public are not aware of the existence of the service [56,71]. With the improvement of awareness, the public would think it is a reasonable choice to use e-consultation and would not make negative assessments on it.

Our results show that personal innovativeness has an effect on behavior intention for the older population but has no effect for the younger population. A study on users not using e-consultation also showed that age had a moderation effect on behavior intention [57]. We think that for the young, especially between the ages from 18 years to 30 years in our study, their innovativeness generally has a high level; therefore, the path loading of personal innovativeness and behavior intention are not significant. Besides, perceived usefulness has less effect on the behavior intention for the older, because with the increase in age, patients place more emphasis on service attitude and medical process and not just utility [72]. Compared with the age factor, the usage experience has only a slight moderation effect on the usage mechanism. The effect direction of the paths is not changed, which indicates that the mechanisms of factors are consistent between the used and unused. Of course, we can also find that personal innovativeness has more effect on the perceived ease of use for the unused. Therefore, raising the innovativeness of the unused might achieve better effect during the promotion of e-consultation.

### Limitations

Our data was collected by a web-based survey; therefore, some selection bias was unavoidable. First, this study showed that 67.5% (630/934) of the respondents were experienced in using e-consultation; however, the usage rate of the students was only 25.7% in our previous survey. These data show that our respondents use mobile devices or computers more frequently. Thus, a higher awareness of e-consultation was observed among these respondents. Second, most respondents came from urban areas (797/934, 85.4%). They might be less willing to use e-consultation because it is easier for them to receive high quality medical resources in the cities. Third, the average age of the respondents was 31 years in our study and 78.3% (731/934) of the respondents had higher education degrees than bachelor’s degree; therefore, our sample may have less medical demand and usage of e-consultation [56]. Besides, the young sample would influence the moderation effect of age. Although it is a better way to survey more people with usage experience through web-based surveys, it is not suitable for all people such as the older adults or the undereducated. Therefore, further offline population-based surveys are necessary, which could be a household survey of residents for small samples with cluster-stratified sampling. In addition, our survey meets the requirements of health care consultations during the COVID-19 pandemic, wherein the public had to stay at home, which might make people have a high intention to use e-consultation.

### Conclusions

Our research focuses on the positive and negative factors that influence the public acceptance of e-consultation and supports the use of TAM and perceived risk in explaining public intention to use e-consultation. We found that perceived usefulness and perceived risk are the most important determinants effecting people’s intention to use e-consultation. Therefore, platforms and manufacturers must improve the function of e-consultation, which will promote the public intention to use it fundamentally. Further, to control the perceived risk of public, government should play an important role in enforcing management of e-consultation markets and approving corresponding medical insurance policies. Besides, we found that personal innovativeness has an effect on behavior intention and the path of factors has differences among people with different characteristics to some degree. Therefore, it is necessary to adjust the strategies to adapt to different groups.

## Appendix

Multimedia Appendix 1 Checklist for Reporting Results of Internet E-Surveys.

## Abbreviations

CHERRIES: Checklist for Reporting Results of Internet E-Surveys
TAM: technology acceptance model
