# Supplementary material for: Perceived Factors Influencing the Public Intention to Use E-Consultation: Analysis of Web-Based Survey Data
Source: J Med Internet Res. 2021 Jan 20;23(1):e21834. doi: 10.2196/21834 (PMC7857952; doi:10.2196/21834)
Supplement: Multimedia Appendix 1 [file jmir_v23i1e21834_app1.docx]

| **Checklist for Reporting Results of Internet E-Surveys (CHERRIES)** | | | |
| --- | --- | --- | --- |
| **Item Category** | **Checklist Item** | **Explanation** | **Where to find it** |
| Design | Describe survey design | Describe target population, sample frame. Is the sample a convenience sample? (In “open” surveys this is most likely.) | See the Methods section, subsection Data Collection in Page 9-10. “A Web-based survey . . . reached the inclusion criteria of survey.” |
| IRB (Institutional Review Board) approval and informed consent process | IRB approval | Mention whether the study has been approved by an IRB. | See the Methods section, subsection Data Collection in Page 10. “The study was approved by the ethics committee of Capital Medical University (number Z2019SY017).” |
|  | Informed consent | Describe the informed consent process. Where were the participants told the length of time of the survey, which data were stored and where and for how long, who the investigator was, and the purpose of the study? | See the Methods section, subsection Data Collection in Page 10. “At the beginning of the electric questionnaire, the following information was given firstly: the purpose of the project; information and instructions regarding the questionnaire; assurance of proper handling of personal information, and the names of research institution.” |
|  | Data protection | If any personal information was collected or stored, describe what mechanisms were used to protect unauthorized access. | All questionnaires were completed after respondents’ login and online filling, and all data were automatically collected in the platform background. The first author of this study can see these data after registering the account and logging in the website (Sojump). |
| Development and pre-testing | Development and testing | State how the survey was developed, including whether the usability and technical functionality of the electronic questionnaire had been tested before fielding the questionnaire. | See the Methods section, subsection Study Design in Page 8. “All survey items . . . from Chinese to English by another qualified translator.” |
| Recruitment process and description of the sample having access to the questionnaire | Open survey versus closed survey | An “open survey” is a survey open for each visitor of a site, while a closed survey is only open to a sample which the investigator knows (password-protected survey). | Our survey is open for each visitor. |
|  | Contact mode | Indicate whether or not the initial contact with the potential participants was made on the Internet. (Investigators may also send out questionnaires by mail and allow for Web-based data entry.) | The initial contact with the potential participants was made on the Internet |
|  | Advertising the survey | How/where was the survey announced or advertised? Some examples are offline media (newspapers), or online (mailing lists – If yes, which ones?) or banner ads (Where were these banner ads posted and what did they look like?). It is important to know the wording of the announcement as it will heavily influence who chooses to participate. Ideally the survey announcement should be published as an appendix. | The survey was announced in the Sojiangwang(www.sojiang.com). The Sojiangwang is a platform that all kinds of people could register in. The Sojianwang would ask every registrant to uploading the real identity information and audit the identity information. In this platform, the registrant could see all different questionnaires when they meet the inclusion standard of questionnaires. All different questionnaires would been named with a unified format: “questionnaire + number” in Sojiangwang. |
| Survey administration | Web/E-mail | State the type of e-survey (eg, one posted on a Web site, or one sent out through e-mail). If it is an e-mail survey, were the responses entered manually into a database, or was there an automatic method for capturing responses? | See the Methods section, subsection Data Collection in Page 10. “The questionnaire link provided on the website (Sojiangwang) can not be copied. After users fill in the questionnaire through the link, the link will be removed from the list and cannot be used repeatedly.” And there was an automatic method for capturing responses by the platform. |
|  | Context | Describe the Web site (for mailing list/newsgroup) in which the survey was posted. What is the Web site about, who is visiting it, what are visitors normally looking for? Discuss to what degree the content of the Web site could pre-select the sample or influence the results. For example, a survey about vaccination on a anti-immunization Web site will have different results from a Web survey conducted on a government Web site | The survey was announced in the Sojiangwang (www.sojiang.com). The Sojiangwang is a platform that all kinds of people could register in. The Sojianwang would ask every registrant to uploading the real identity information and audit the identity information. In this platform, the registrant could see all different questionnaires when they meet the inclusion standard of questionnaires. All different questionnaires would been named with a unified format: questionnaire + number in Sojiangwang. |
|  | Mandatory/voluntary | Was it a mandatory survey to be filled in by every visitor who wanted to enter the Web site, or was it a voluntary survey? | Voluntary |
|  | Incentives | Were any incentives offered (eg, monetary, prizes, or non-monetary incentives such as an offer to provide the survey results)? | prizes incentives |
|  | Time/Date | In what timeframe were the data collected? | See the Methods section, subsection data collection in Page 10. “The data was collected from March 30th 2020 to April 4th 2019.” |
|  | Randomization of items or questionnaires | To prevent biases items can be randomized or alternated. | There was no randomization |
|  | Adaptive questioning | Use adaptive questioning (certain items, or only conditionally displayed based on responses to other items) to reduce number and complexity of the questions. | Adaptive questioning was used for on questions (item 9 of part 1) where follow-up items were displayed conditionally with a confirmative answer |
|  | Number of Items | What was the number of questionnaire items per page? The number of items is an important factor for the completion rate. | Number of questions page 1 varied widely from 1 to 10.  Number of questions page 2 varied widely from 1 to 38 (Questionnaire matrix). |
|  | Number of screens (pages) | Over how many pages was the questionnaire distributed? The number of items is an important factor for the completion rate. | The number of screens was 2 |
|  | Completeness check | It is technically possible to do consistency or completeness checks before the questionnaire is submitted. Was this done, and if “yes”, how (usually JAVAScript)? An alternative is to check for completeness after the questionnaire has been submitted (and highlight mandatory items). If this has been done, it should be reported. All items should provide a non-response option such as “not applicable” or “rather not say”, and selection of one response option should be enforced. | There were checks for completeness before the questionnaire has been submitted. The platform would remind the respondents by highlighting mandatory items. Only the questionnaire completed could be submit. |
|  | Review step | State whether respondents were able to review and change their answers (eg, through a Back button or a Review step which displays a summary of the responses and asks the respondents if they are correct). | The respondents were able to review and change their answers through a Back button before they submit the questionnaire. |
| Response rates | Unique site visitor | If you provide view rates or participation rates, you need to define how you determined a unique visitor. There are different techniques available, based on IP addresses or cookies or both. | We did not provide view rates or participation rates |
|  | View rate (Ratio of unique survey visitors/unique site visitors) | Requires counting unique visitors to the first page of the survey, divided by the number of unique site visitors (not page views!). It is not unusual to have view rates of less than 0.1 % if the survey is voluntary. | We did not provide view rate. |
|  | Participation rate (Ratio of unique visitors who agreed to participate/unique first survey page visitors) | Count the unique number of people who filled in the first survey page (or agreed to participate, for example by checking a checkbox), divided by visitors who visit the first page of the survey (or the informed consents page, if present). This can also be called “recruitment” rate. | We did not provide participation rate. |
|  | Completion rate (Ratio of users who finished the survey/users who agreed to participate) | The number of people submitting the last questionnaire page, divided by the number of people who agreed to participate (or submitted the first survey page). This is only relevant if there is a separate “informed consent” page or if the survey goes over several pages. This is a measure for attrition. Note that “completion” can involve leaving questionnaire items blank. This is not a measure for how completely questionnaires were filled in. (If you need a measure for this, use the word “completeness rate”.) | We did not provide Completion rate. |
| Preventing multiple entries from the same individual | Cookies used | Indicate whether cookies were used to assign a unique user identifier to each client computer. If so, mention the page on which the cookie was set and read, and how long the cookie was valid. Were duplicate entries avoided by preventing users access to the survey twice; or were duplicate database entries having the same user ID eliminated before analysis? In the latter case, which entries were kept for analysis (eg, the first entry or the most recent)? | See the Methods section, subsection Data Collection in Page 10. “The Sojianwang would ask every registrant to uploading the real identity information and audit the identity information. In this platform, the registrant could see all questionnaires when they meet the inclusion standard of questionnaires.” “The questionnaire link provided on the website (Sojiangwang) can not be copied. After users fill in the questionnaire through the link, the link will be removed from the list and cannot be used repeatedly.”  And the visitors of Sojiangwang only see the “questionnaire + number”on the web pages. If they want to know about the content of the questionnaire, they must enter the link. If they don’t want to fill, they can drop out but not enter again. |
|  | IP check | Indicate whether the IP address of the client computer was used to identify potential duplicate entries from the same user. If so, mention the period of time for which no two entries from the same IP address were allowed (eg, 24 hours). Were duplicate entries avoided by preventing users with the same IP address access to the survey twice; or were duplicate database entries having the same IP address within a given period of time eliminated before analysis? If the latter, which entries were kept for analysis (eg, the first entry or the most recent)? | See above |
|  | Log file analysis | Indicate whether other techniques to analyze the log file for identification of multiple entries were used. If so, please describe. | n / a |
|  | Registration | In “closed” (non-open) surveys, users need to login first and it is easier to prevent duplicate entries from the same user. Describe how this was done. For example, was the survey never displayed a second time once the user had filled it in, or was the username stored together with the survey results and later eliminated? If the latter, which entries were kept for analysis (eg, the first entry or the most recent)? | See above |
| Analysis | Handling of incomplete questionnaires | Were only completed questionnaires analyzed? Were questionnaires which terminated early (where, for example, users did not go through all questionnaire pages) also analyzed? | We only included the questionnaires that were completed. |
|  | Questionnaires submitted with an atypical timestamp | Some investigators may measure the time people needed to fill in a questionnaire and exclude questionnaires that were submitted too soon. Specify the timeframe that was used as a cut-off point, and describe how this point was determined. | The cut-off point is <100 seconds and >1000 seconds. We counted the time of undergraduates to fill in the questionnaire for the first time, which was about 5 minutes. We used the standard of three times to determine the timeframe. Meanwhile, we found many respondents take 900-1000 seconds to fill. But the respondents who spent longer than 1000 seconds to fill in all are single case. So we excluded the respondents using >1000 seconds |
|  | Statistical correction | Indicate whether any methods such as weighting of items or propensity scores have been used to adjust for the non-representative sample; if so, please describe the methods. | n / a |
